# Validation of an improved helical diode array and dose reconstruction software using TG‐244 datasets and stringent dose comparison criteria

**DOI:** 10.1120/jacmp.v17i6.6414

**Published:** 2016-11-08

**Authors:** Saeed Ahmed, Benjamin Nelms, Jakub Kozelka, Geoffrey Zhang, Eduardo Moros, Vladimir Feygelman

**Affiliations:** ^1^ Department of Physics University of South Florida Tampa FL USA; ^2^ Canis Lupus LLC Merrimac WI USA; ^3^ Sun Nuclear Corp., Melbourne FL USA; ^4^ Department of Radiation Oncology, Moffitt Cancer Center Tampa FL USA

**Keywords:** IMRT QA, VMAT QA, measurement‐guided dose reconstruction, three‐dimensional dosimetry, diode array

## Abstract

The original helical ArcCHECK (AC) diode array and associated software for 3D measurement‐guided dose reconstruction were characterized and validated; however, recent design changes to the AC required that the subject be revisited. The most important AC change starting in 2014 was a significant reduction in the overresponse of diodes to scattered radiation outside of the direct beam, accomplished by reducing the amount of high‐Z materials adjacent to the diodes. This change improved the diode measurement accuracy, but in the process invalidated the dose reconstruction models that were assembled based on measured data acquired with the older version of the AC. A correction mechanism was introduced in the reconstruction software (3DVH) to accommodate this and potential future design changes without requiring updating model parameters. For each permutation of AC serial number and beam model, the user can define in 3DVH a single correction factor which will be used to compensate for the difference in the out‐of‐field response between the new and original AC designs. The exact value can be determined by minimizing the dose‐difference with an ionization chamber or another independent dosimeter. A single value of 1.17, corresponding to the maximum measured out‐of‐field response difference between the new and old AC, provided satisfactory results for all studied energies (6X, 15X, and flattening filter‐free 10XFFF). A library of standard cases recommended by the AAPM TG‐244 Report was used for reconstructed dose verification. The overall difference between reconstructed dose and an ion chamber in a water‐equivalent phantom in the targets was 0.0% ± 1.4% (1 SD). The reconstructed dose on a homogeneous phantom was also compared to a biplanar diode dosimeter (${\text{Delta}}^{4}$) using gamma analysis with 2% (local dose‐error normalization)/2 mm/10% cutoff criteria. The mean agreement rate was 96.7% ± 3.7%. For the plans common with the previous comparison, the mean agreement rate was 98.3% ± 0.8%, essentially unchanged. We conclude that the proposed software modification adequately addresses the change in the dosimeter response.

PACS number(s): 87.55Qr

## I. INTRODUCTION

The ArcCHECK (AC), developed by Sun Nuclear Corp., Melbourne, FL, is a diode‐array dosimeter used primarily for quality assurance of IMRT/VMAT treatments. It can be employed by itself to compare measured and reference dose at the detectors’ locations^1^, ^2^, ^3^, ^4^, ^5^, ^6^ or in conjunction with 3DVH software (Sun Nuclear) that uses a AC‐based planned dose perturbation (ACPDP) algorithm to perform volumetric measurement‐guided dose reconstruction on a phantom or patient dataset.^7^, ^8^, ^9^, ^10^, ^11^ While the dosimeter and associated software have been characterized and employed rather extensively,^(1–3,5,7,9,10,12–18)^ two recent developments necessitated revisiting the subject.

First, there have been several design changes in the AC hardware since the original calibration factors^(5)^ and 3DVH beam models^(7)^ were validated. These changes were implemented to improve device performance and regulatory compliance, but it was suspected that some of the changes could influence the dosimeter response characteristics, which would naturally change the volumetric dose reconstruction with 3DVH. The reconstruction procedure was optimized based on the original AC design. It is good practice to repeat the basic characterization of the array, to rule out (or mitigate if necessary) any unexpected effects and verify the intended improvements. In addition, while the practical use of the AC with 10 MV flattening filter‐free beam has been reported,^(19)^ the comprehensive evaluation has not.

Second, in departure from the previous school of thought,^(20)^ the AAPM TG‐244 Report on medical physics practice guidelines for commissioning of dose calculations^(21)^ allows the use of electronic dosimetry arrays as a primary commissioning tool for evaluating IMRT/VMAT dose distributions, provided that dose measurement or reconstruction is performed with adequate spatial resolution. Furthermore, TG‐244 makes challenging, patient‐based cases (image sets, structure sets, and detailed lists of dosimetric objectives) available for download to be used for commissioning of the IMRT/VMAT systems. It would be useful for the clinical physics community to have a benchmark of the level of dosimetric agreement achievable with the AC and 3DVH for these cases. Unlike the previously reported TG‐119 datasets,^(22)^ the TG‐244 ones are more clinically relevant and include fairly large target volumes, which are known to be challenging for the TPS calculation accuracy. Finally, TG‐244 suggests more stringent dose comparison metrics be employed in addition to the ubiquitous^20^, ^23^ 3% (global dose‐error normalization) / 3 mm distance‐to‐agreement gamma passing rates that were used in the TG‐119 report,^(22)^ but since then have been largely proven to be neither sensitive nor specific.^(18,24–28)^

## II. MATERIALS AND METHODS

### A. General

All measurements were performed with conventional (6X and 15X) and flattening filter‐free 10X (10XFFF) beams from a TrueBeam v 2.0 linear accelerator equipped with a 120‐leaf Millennium multileaf collimator (MLC) from Varian Medical Systems, Palo Alto, CA. The treatment planning system (TPS) was Pinnacle v. 9.8 (Philips Radiation Oncology Systems, Fitchburg, WI).

The AC features an array of 1,386 point detectors that form a 10.4 cm radius cylindrical active surface inside a doughnut‐shaped PMMA phantom. The phantom has an outer diameter of 26.6 cm and a 15 cm diameter inner hole that can be plugged with a PMMA cylinder.^(5)^ All tests in this work were performed with the plug inserted, as this is the configuration necessary for 3D dose reconstruction.^(7)^ The phantom was represented in the TPS by a cylinder with a uniform relative density of 1.15, which was shown previously to provide adequate agreement between the ion chamber measurements and TPS calculations.^(5)^

ArcCHECK measurement data were collected using SNC Patient software v. 6.6 (Sun Nuclear Corp). Statistical analyses were performed with GraphPad Prism software package (v. 6, GraphPad Software, La Jolla, CA).

### B. ArcCHECK hardware changes

The original AC is designated by the manufacturer as Version 1, while the redesigned one, subject of the current investigation, is Version 2. Version 2 began shipping in 2014, in part to meet the European Union hazardous substances mandate.^(29)^ One difference between the “old” (v.1) and “new” (v.2) AC hardware is a redesign of the readout electronics. An onboard buffer memory, in combination with a more robust interface and connection cable, ensures better reliability of 50 ms updates recording. The new electrometers are more sensitive and also more stable with respect to zero drift, both features particularly advantageous for longer and/or low dose rate measurements.

The second major change — the one that directly affects the dosimeter response — was a reduction in the overall amount of high‐atomic‐number materials, particularly lead, used in diode and printed circuit board manufacturing. Also, wherever possible, metal conductors were placed farther away from the sensitive volumes. Since the energy dependence of the diode is largely determined by the surroundings of the die,^(30)^ the impact on detector response had to be thoroughly investigated.

### C. Basic dosimeter properties

A full dosimetric characterization of the new AC was performed, as warranted by significant design changes. However, for brevity we will only elaborate here on those tests that have not been performed before either at all or for certain energies, or produced results substantially different from the v. 1 AC.

#### C.1 Dosimetric tests with no substantial new findings

The tests in this category included: a classic relative calibration test (the 180° “detector fip”);^3^, ^31^ a diode sensitivity as a function of field size test; and diode sensitivity as a function of position in transverse and longitudinal directions tests. In all cases the device performed within specifications and/or substantially equivalent to the previous version.^4^, ^5^, ^14^

#### C.2 Dose and dose‐rate dependencies

The AC diodes are potentially subject to a variety of sensitivity changes related to both the accumulated dose and dose rate, as described below.

##### C.2.1 Low MU linearity

With the central plug in place, the AC exit dose is about one‐quarter of the entrance one. Therefore the dose linearity has to be studied separately for different diode positions. The relative AC response for the entrance and exit diodes in a $10\times 10\;{\text{cm}}^{2}$ 6X beam was studied in the 1–100 MU range against the ion chamber. Because of the known long‐lived trap population effect,^(32)^ these measurements were performed with two SNC Patient software settings. At first the default parameters were used, whereby the beam‐on recognition was turned on and 10 measurement updates (every 50 ms) were collected after the beam‐off flag. In the subsequent measurement series, effectively all the updates were collected, whether the beam was on or off.

##### C.2.2 Dose per pulse

Diode sensitivity variation with dose per pulse (DPP) was measured against the IC by varying the source‐to‐detector distance (SDD). Within the studied SDD range from 74.6 to 119.6 cm, the dose per pulse changed from approximately $2\times 10^{-4}$ to $5\times 10^{-4}$ Gy for the 6X beam and from $2\times 10^{-3}$ to $6\times 10^{-3}$ Gy for 10XFFF. The AC or the dummy shell with the IC were irradiated by a vertical beam and the readings were taken at the entrance (average of X=±5 mm for the AC). The normalized ratios of AC dose to IC reading were recorded.

##### C.2.3 Repetition rate

The accelerator repetition rate (MU/min) dependence was studied less frequently than dose per pulse one. However, such dependence for the AC diodes does exist^(5)^ and was hypothesized by Jursinic^(32)^ to be attributable to the capture of excess minority carriers by charge traps. The slow reopening of these traps should occur on the hundreds of milliseconds to seconds time scale to serve as the physical basis for the repetition rate dependence. The repetition rate was varied from 5 to 600 MU/min for the 6X beam and from 400 to 2400 Mu/min for 10XFFF, extending the repetition rate range compared to the previous reports.^4^, ^5^ The diode doses were divided by the corresponding IC readings, and normalized at 400 MU/min, a common repetition rate setting for both X‐ray energies.

#### C.3 Out‐of‐field sensitivity

Being not water‐equivalent, diode detectors have energy dependence, and one of the manifestations of this fact is a variation in sensitivity with the distance from the aperture edge outside the direct beam, due to the energy spectrum changes. The change in sensitivity to scattered low‐energy photons is the largest difference between the two AC designs that is apparent to the end user. To quantify the changes, the diode‐loaded AC (v.1 and then v.2) and the dummy AC shell with the ion chamber were aligned at the isocenter and then slightly rotated to place the sensitive volume at the central axis. A series of the MLC apertures were designed. They varied from 0.5 to 3 cm in width and were positioned such that the measurement point was either 1 or 2 cm outside the field. The AC diode and the IC readings were normalized to their respective open $10\times 10\;{\text{cm}}^{2}$ fields values, and the AC/IC ratios were plotted for the 6X and 15X beams.

### D. Three‐dimensional dose reconstruction

#### D.1 3DVH software changes

##### D.1.1 Background

3DVH software uses time‐resolved dose information from the AC to generate high density 3D dose grids. Necessary to this process is a library of beam models, each of which is unique for specific linac/energy/MLC combinations.^(7)^ These models are essential to create a high density, high resolution dose grid for each subbeam, that can then be perturbed to fit the corresponding measurements at the entry and exit surfaces.^(7)^ The model library was developed based on measurements from the AC v.1. The AC data acquisition software currently does not include an explicit correction for the diode out‐of‐field (OOF) overresponse. Therefore this overresponse was implicitly accounted for in the volumetric reconstruction model, to ensure favorable comparison with the independent IC measurements inside the volume. Consequently, if/when the OOF sensitivity changes with new hardware designs, the method's original compensation for overresponse is no longer optimized and would lead to erroneous results. While it is theoretically possible to create different model versions that are specific to each new AC hardware configuration, this would be prohibitive from the product maintenance standpoint. Instead, the strategy was to keep the existing, universal library of beam models and provide a simple correction mechanism to tune it for a particular AC version. A correction factor can now be assigned by the user for each distinct combination of the AC hardware version (identified by serial number) and 3DVH beam model. This correction factor is a single, relative value that essentially quantifies the difference in response to the OOF radiation between the new original AC designs. The default correction of 1.0 means “no change”, but for any other value, a special correction takes place during the reconstruction of each time‐resolved subbeam's absolute dose. Taking the current AC design change as an example, the beam model that compensated for the original overresponse now overcompensates. Left uncorrected, ACPDP dose would be systematically low even though the measurement data are technically more accurate. The purpose of the correction is simply to dampen the compensation and “add back” small amounts of dose, but only in the areas of each subbeam that are under an MLC leaf and/or jaw.

##### D.1.2 Correction method

The straightforward solution would be to split the 3D dose into primary (i.e., transmission) and scatter components for every reconstructed subbeam, then correct only the scatter component. We implemented this method at first, only to realize that it caused a marked increase in the dose reconstruction time. Therefore, we implemented a simplified and fast method. This approach creates a single 2D correction mask for each reconstructed subbeam, where the mask is in a beam's‐eye‐view (BEV) sense perpendicular to the beam axis, following divergent lines from the source. The correction mask is constant over all depths but tapered to unity at the curved entry and exit surfaces of the diode array. The dose reconstruction time using the simplified method was, on average, within 10% of the original (i.e., uncorrected) calculation time.

The dose correction factor for any point (x,y,z) in the beam coordinate system for a modulated subbeam is summarized by the equations below.

(1) $$\begin{array}{r} {\text{Dose}}_{sub–beam,corrected}(x,y,z)={\text{Dose}}_{sub–beam,uncorrected}{\left(x,y,z\right)}^{*}\\ \;Composite\;OOF\;Correction(x,y) \end{array}$$

where *x* and *y* are the ray position coordinates in BEV and *z* is the orthogonal (depth) coordinate. The last term in Eq. (1) is defined as

(2) $$Composite\;OOF\;Correction\;(x,y)=\frac{\int_{MU_{start}}^{MU_{end}}[Corrected\;Fluence\;(x,y,MU)\;dMU]}{\int_{MU_{start}}^{MU_{end}}[Uncorrected\;Fluence\;(x,y,MU)\;dMU]}$$

The *MU* parameter represents the dynamic progression for modulated beams derived from the meterset progression in the DICOM RT Plan. Integration (summation) is performed from start to end of each subbeam. *Uncorrected Fluence* for any *x, y* point is a measure of “exposure” to the beam, or cumulative time a point spends inside an open aperture (1.0 contribution) relative to being under an MLC leaf (contribution equals MLC transmission fraction). This value is then corrected:

(3) $$\begin{array}{rl} & Corrected\;Fluence\;(x,y,MU)=Uncorrected\;Fluence\;(x,y,MU)+\\ & \;OOF\;Adjustment\;(x,y,MU) \end{array}$$

In turn,

(4) $$\begin{array}{rl} & OOF\;Adjustment\;(x,y,MU)\\ & \;=\left\{ \begin{array}{l} {\left(Nominal\;OOFCF–1.0\right)}^{*}\;EDF,\;\;for\;regions\;under\;collimation\\ 0.0,\;\;for\;directly\;irradiated\;regions,i.e.\;inside\;the\;momentary\;aperture \end{array}\right. \end{array}$$

where *Nominal OOFCF* is the nominal out‐of‐field correction factor (specified by the user on the software setup tab) and *EDF* is the energy dependence factor, a small fraction derived from the PDP model parameters. The EDF increases with energy but is always a small number, on the order of 0.01 to 0.025. This factor is introduced so that the user‐defined Nominal OOFCF stays effectively the same across all energies, for ease‐of‐use.

#### D.2 ACPDP comparisons with direct measurements

##### D.2.1 Test plans

IMRT and VMAT plans were selected based on the TG‐244 Report recommendations.^(21)^ They included two well‐known plans from the TG‐119 suite^(22)^ (C‐shape and Mock Head and Neck (H&N), optimized on the $20\times 20\times 20\;{\text{cm}}^{3}$ plastic water (PW) Cube Phantom from CIRS Inc., Norfolk, VA), and three more realistic plans from the downloadable datasets in the TG‐244 library:^(21)^ Anal, H&N, and Abdomen. The concept behind the TG‐244 cases is to provide challenging but clinically relevant goals, with large targets and tight constraints, resulting in highly modulated plans pushing the accuracy limits of the TPS calculation algorithms. These datasets were previously used for large, inter‐institutional plan studies similar to the pilot study described by Nelms et al.^(33)^

All plans were optimized based on 6X beams and for the purpose of this work simply recalculated for other energies.^(21)^ All plans were created with both VMAT (Pinnacle SmartArc) and step‐and‐shoot IMRT (Pinnacle Direct Machine Parameters Optimization) techniques. The TG‐244 H&N and Anal VMAT plans used two arcs and the rest one. The VMAT plans were calculated with 2° angular control points (CP) increment. The IMRT plans used seven to nine equidistant gantry angles. Two of the plans (TG‐244 H&N and Anal) had targets too large to be encompassed for conventional IMRT with a single set of Varian MLC carriage positions, due to the limitations of the MLC leaf extension. They were instead planned with the “wide‐field” IMRT technique, where the leaves are allowed to nearly close inside the treatment field, not necessarily under the X jaws, but those leaf abutment points move across the field from segment to segment, to avoid excessive exposure at any one location in the patient. In all cases, uniform 2.5 mm dose grid resolution was used.

##### D.2.2 ACPDP point dose comparisons

Point doses in the high‐dose, low‐gradient regions were measured in the PW Cube Phantom with a $0.125\;{\text{cm}}^{3}$ Model TN31010 ion chamber (PTW, Freiburg, Germany). The chamber was cross‐calibrated in a $10\times 10\;{\text{cm}}^{2}$ field against the expected TPS dose prior to every measurement session. The chamber volume was drawn as a region of interest and the corresponding mean dose was used for comparisons. The confidence limit (CL) of the average difference between the measured and reconstructed dose was expressed as mean ±1.96 standard deviation.^22^, ^34^ Note that this confidence limit is different (larger) than the traditional statistical confidence interval.

##### D.2.3 ACPDP dose distribution comparisons with a biplanar array

The ACPDP volumetric dose was directly compared to the applicable measurement planes of a previously validated biplanar dosimeter.^35^, ^36^, ^37^, ^38^, ^39^ Excellent agreement between ACPDP reconstructed dose (based on the original AC design) and ${\text{Delta}}^{4}$ (ScandiDos AB, Uppsala, Sweden) measurements was demonstrated earlier.^(10)^ Note that the ${\text{Delta}}^{4}$ calibration formalism includes an adequate empirical software correction for the diode response variation outside the beam aperture.^(37)^ It is however a second‐order correction for the cumulative dose measurements.

The AC phantom with the plug was centered on the lasers. The daily correction factor was determined by comparing measured and calculated doses in the central portion of the parallel‐opposed $10\times 10\;{\text{cm}}^{2}$ field pair, at the midrange repetition rate.^5^, ^32^ Three‐dimensional ACPDP dose reconstruction was performed with a prototype version of 3DVH software incorporating OOF corrections as described above. The cylindrical ${\text{Delta}}^{4}$ phantom represented the “patient” dataset used for dose reconstruction.^(10)^ The reconstructed dose grid was saved in DICOM RT Dose format and imported into the ${\text{Delta}}^{4}$ software as reference dose. After the treatment was delivered to the ${\text{Delta}}^{4}$ in a usual fashion, the reconstructed dose was compared to direct measurement using built‐in gamma analysis tools.^(40)^ Both 3%/2 mm and 2%/2 mm criteria combinations were used, with global (e.g., 3% G) and local (e.g., 3% L) dose‐error normalization.

Low dose analysis threshold was 10% of the maximum. For conciseness, only the results with 2% dose‐error criteria are presented in detail when comparing the AC to the ${\text{Delta}}^{4}$.

##### D.2.4 Comparisons with the TPS

One of the goals of this paper, in addition to validating the modified ACPDP hardware and software, is to estimate the range of achievable gamma analysis results on a phantom for the TG‐244 datasets, which unlike TG‐119,^(22)^ have not been extensively studied so far. To that end, we present the data for both the direct AC to TPS comparison and the comparison between the 3D ACPDP and TPS dose grids. In this case, the results of gamma analysis with 3% G/2 mm are also included, as this seems to be the emerging criteria combination for patient‐specific IMRT QA. Following the TG‐119 methodology,^(22)^ the 95% one‐sided confidence CL is established for the gamma analysis passing as 100% ‐ (mean passing rate) ‐ 1.96*(standard deviation).^(22)^

## III. RESULTS AND DISCUSSION

### A. Basic dosimeter properties

#### A.1 Dose and dose rate dependencies

##### A.1.1 Low MU linearity

**Figure 1:**
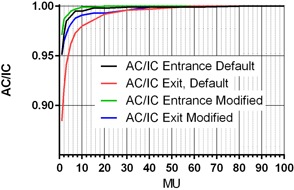
Low MU nonlinearity. AC exit and entrance doses are normalized to the ion chamber and shown as a function of MU. The default software settings limit the number of updates after the beam is turned off, while the modified settings do not.

The results are presented in Fig. 1. The deviation from the straight line for the 2 MU setting (entrance diode) is −2.8%, somewhat larger than the −0.9% error reported by Li et al.^(14)^ for the comparable data point with the AC v.1. Since for the same MU setting the exit dose is about one‐quarter of the corresponding entrance one, the apparent nonlinearity of response is more pronounced for the exit diodes. Extending the integration time (modified vs. default settings curves in Fig. 1) reduces the maximum nonlinearity by approximately one‐half, consistent with the notion that shorter integration time prevents all the charge trapped in the long‐lived centers from being collected.

The residual nonlinearity is likely associated with the intrinsic diode properties^(32)^ rather than the readout electronics, since similar effects were reproduced with this type of diode connected to a variety of electrometers in different devices. It was also verified by the ion chamber measurements at the different depths in a phantom that the observed effect is not caused by the possible initial beam instability during short exposures. The investigated version of AC (v.2) has a more robust background and zero‐drift suppression that its predecessor, and therefore the modified settings with long integration times were used for subsequent data collection. Longer integration times should become the default for the AC v.2. Current findings agree with the AC specifications of <0.5% integration non‐linearity from 6 to 30 cGy total dose.^(41)^ However, it must be noted that, for the 6X beam, the exit dose with the plug is approximately 0.3 cGy/MU and thus the specifications do not address dose linearity for segments with less than ∼20 MU, which are quite common with standard fractionation. The results in Fig. 1 provide the nonlinearity estimates down to 1 MU.

##### A.1.2 Dose per pulse

Within the studied SDD range from 74.6 to 119.6 cm, the AC/IC ratio varied smoothly from 1.012 to 0.992, and from 1.001 to 0.995 for the 6X and 10X FFF beams, respectively. The ratios were normalized to 1.000 at the calibration SDD of 89.6 cm. The overall change in response of ∼2% for the 6X beam is substantially smaller than reported by Chaswal et al.^(4)^ Their 7% variation for the same SDD range, based on the inverse‐square dose estimates, appears excessive compared to the previous reports studying similar diodes.^14^, ^31^, ^42^ We believe that the methodology of direct comparison with recombination‐corrected IC employed in this work is a well‐established standard for these types of measurements and provides a more realistic estimate of the SDD dependence, in line with the previous findings. With the 10X FFF dose per pulse being approximately an order of magnitude higher than 6X, the large percentage of the charge traps remain populated regardless of the SDD within the studied limits, and there was little change in diode sensitivity.

##### A.1.3 Repetition rate

**Figure 2:**
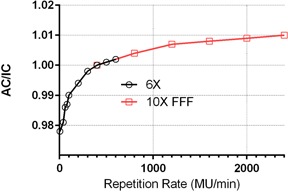
Repetition rate dependence for the 6X and 10X FFF beams. The AC/IC ratio is normalized at 400 MU/min.

The AC response relative to the IC as a function of repetition rate is presented in Fig. 2. Normalized at 400 MU / min, the curve is relatively smooth, with two different energies used for the low and high end of the repetition rate range. For the lowest (5 MU / min) repetition rate the AC/IC ratio was 0.978, which is reasonably close to the previously reported value of 0.986.^(5)^ The difference did not exceed 1% for the 100–600 MU/min range with the 6X beam and the entire 400–2400 MU/min range with the 10X FFF beam.

#### A.2 Out‐of‐field sensitivity

**Figure 3:**
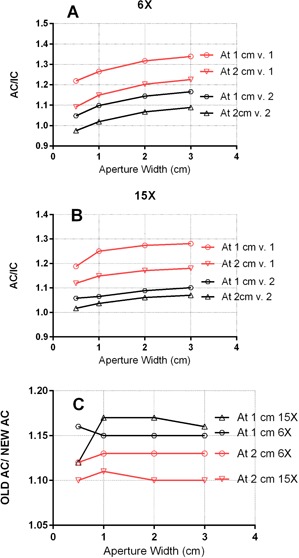
Ratios of readings at the points 1 and 2 cm outside the field as a function of the aperture width: AC to IC ratios for (a) the old (v.1); (b) the new (v.2) versions of the ArcCHECK; (c) the ratios of the v.1 to v.2 AC readings.

Normalized ratios of the AC to IC readings outside the field are presented in Figs. 3(a) and 3(b). Since the amount of scattered radiation reaching the point of measurement increases with the field size and decreases with the distance to the field edge, it is natural that the relative sensitivity of the diode would generally increase as shown in Fig. 3. With the exception of one data point, the ratio of AC to IC stays fairly flat for the given hardware version, namely within 1%. It is clear from Fig. 3 that the AC v.2 has substantially lower overresponse outside the field, which is consistent with the reduction in the amount of high‐Z materials present in the newer design. In comparison with the previous work,^(4)^ we believe that directly referencing the ion chamber provides a more realistic overresponse estimate than their TPS‐based analysis. The out‐of‐field response was also reported in 2013, presumably for the v. 1 of the AC, by Li et al.^(14)^ The differences in the geometry preclude detailed comparisons of the results, but under the most similar conditions, our factor for the AC v. 1 appears to be about 10% higher.

The OOF sensitivity change is a second‐order effect which has limited practical consequences when the dosimeter is used to directly compare the diode dose to the TPS. However, it becomes important when the apparent AC dose profiles are used to determine the dose‐spread kernel parameters for internal calculations during volumetric dose reconstruction. The results above clearly support the need for the additional correction factor in the 3D dose‐reconstruction software.

### B. Three‐dimensional dose reconstruction

#### B.1 Selection of the overresponse correction factor in the software

**Figure 4:**
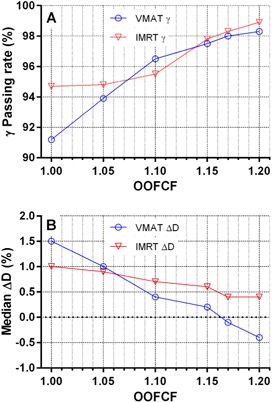
Gamma analysis passing rates (a) (2% L/2) and median dose‐differences (b) for 6X IMRT and VMAT plans, comparing ACPDP with the ${\text{Delta}}^{4}$ as a function of user‐selectable out‐of‐field correction factor (OOCF).

The ratio of the old to new AC diode overresponse from Fig. 3 varied from 1.0 to 1.17 between all geometries and the two extreme energies (6X and 15X). In the search of the simplest practical solution, we elected to use the highest value (1.17) as the starting point, the rationale being that the points with the largest amount of scatter are likely to have the highest OOF absolute dose and thus have the most influence on the final results. This value was further refined by varying the correction factor to maximize the 2% L/2 gamma analysis agreement rate and minimize the median dose deviation for one dataset (TG‐119 C‐shape) between the ACPDP on the ${\text{Delta}}^{4}$ phantom and direct ${\text{Delta}}^{4}$ measurements. This was done for both 6X plans, VMAT and IMRT As one can see in Fig. 4, the median dose difference is minimized around the OOFCF of 1.17, while the passing rates nearly plateau. Since the maximum measured value was 1.17, it was logical not to exceed that value.

#### B.2 ACPDP comparisons with direct measurements

##### B.2.1 Point‐dose comparisons

The average difference for all plans and energies between the ACPDP dose reconstructed on the PW Cube and the ion chamber measurement in the high‐dose low‐gradient regions was 0.0% ± 1.4% (95% CL−2.7% to 2.7%). The distribution passed the D'Agostino & Pearson omnibus normality test and the mean was not statistically significantly different from 0 (*t*‐test p=1.0).

For practical implementation of the modified software with the wider user base, whereby an independent array dosimeter may not be readily available, similar ion chamber measurements are likely to be the primary means of establishing the correction factor. They can be performed in either an arbitrary phantom, as was done here, or with the standard single‐hole PMMA plug in the AC phantom, or with the optional Multi‐Plug that provides a variety of possible chamber locations throughout the reconstruction volume.^(11)^

##### B.2.2 Comparisons with a biplanar array

The detailed results are presented in Table 1. The overall mean agreement rate at the most stringent level of 2% L/2 gamma analysis is 96.7% ± 3.7% (1 SD), which appears somewhat lower than reported with the previous AC/3DVH version (98.2% ± 1.6%).^(10)^ However, a closer look reveals that for the plans common to both investigations (TG‐119 C‐shape and H&N), the mean agreement rate for all energies and techniques is 98.3% ± 0.8%, essentially unchanged from the previous report. Likewise, the TG‐244 Abdomen plan passing rate is 98.5% ± 1.6%. The lower agreement rates that pull down the average are associated only with the larger targets (TG‐244 H&N and Anal plans), particularly with the 10X FFF beam. The worst agreement at the 2% L/2 level is observed for the TG‐244 H&N plans with 10X FFF. With both VMAT and IMRT, the median dose difference between the ACPDP and ${\text{Delta}}^{4}$ in those cases is 1.3%, the largest in the population. On the other hand, the median dose difference averaged across all plans and energies is 0.0% ± 0.6%, with the 95% CL from −1.2% to 1.2% and the distribution passing the normality test. In conjunction with the IC data presented above, this confirms the choice of a single value for the overresponse correction described in the previous section.

**Table 1:** Gamma analysis passing rates and median dose‐differences for 3D ACPDP on the ${\text{Delta}}^{4}$ phantom vs. ${\text{Delta}}^{4}$ directly measured dose

		$ACPDP\;vs.\;Delta^{4}\gamma$ *Passing Rate (%)*		
*Dataset*	*Energy/Technique*	*Local Dose‐error* 2%/2 mm	*Global Dose‐error* 2%/2 mm	*Median* ΔD *(%)*
Abdomen‐TG 244	6X‐VMAT	100.0	100.0	0.2
	6X‐IMRT	98.2	100.0	0.3
	15X‐VMAT	100.0	100.0	‐0.1
	15X‐IMRT	99.1	99.8	0.0
	10XFFF‐VMAT	95.9	96.6	‐0.9
	10XFFF‐IMRT	97.5	99.2	‐0.5
H&N‐TG 244	6X‐VMAT	96.2	98.3	0.4
	6X‐WFIMRT	96.0	97.9	0.3
	15X‐VMAT	98.7	99.6	‐0.1
	15X‐WFIMRT	99.2	99.9	‐0.1
	10XFFF‐VMAT	84.4	92.7	1.3
	10XFFF‐WFIMRT	87.8	94.4	1.3
Anal‐TG 244	6X‐VMAT	99.1	100.0	‐0.1
	6X‐WFIMRT	94.7	97.1	‐0.1
	15X‐VMAT	98.0	99.0	‐0.6
	15X‐WFIMRT	91.8	96.8	‐0.8
	10XFFF‐VMAT	93.3	98.5	0.7
	10XFFF‐WFIMRT	91.1	96.7	0.7
C‐shape‐TG 119	6X‐VMAT	98.0	99.5	‐0.1
	6X‐IMRT	98.4	99.4	0.4
	15X‐VMAT	98.9	99.8	‐0.3
	15X‐IMRT	99.1	99.2	0.6
	10XFFF‐VMAT	97.3	99.0	‐0.7
	10XFFF‐IMRT	99.8	100.0	‐0.5
H&N‐TG 119	6X‐VMAT	97.5	99.4	‐0.4
	6X‐IMRT	97.3	99.7	0.5
	15X‐VMAT	98.2	99.2	‐0.3
	15X‐IMRT	98.6	99.8	0.4
	10XFFF‐VMAT	97.7	98.8	‐0.4
	10XFFF‐IMRT	99.2	100.0	0.0
	Average	96.7	98.7	0.0
	SD	3.7	1.8	0.6
	Min	84.4	92.7	‐0.9
	Max	100.0	100.0	1.3

Relaxing the criteria to 3% L/2 mm results in the average γ analysis agreement rate between the ACPDP and ${\text{Delta}}^{4}$ of 98.3% ± 2.2%, with only four data points below 95% and none below 92%.

The modification to 3DVH software was definitely necessary. Without it, the average ACPDP vs. ${\text{Delta}}^{4}\;2\%\;L/2$ and 2% G/2 mm agreement rates would have been unacceptable: 73% and 78%, respectively, with a bias of ACPDP dose being too low.

##### B.2.3 Comparisons with the TPS

As a point of reference, the average difference between the TPS and IC was 0.6% ± 0.9% (95% CL −1.2% to 2.4%). The distribution did not pass the normality test (p=0.01) and the mean value is statistically significantly different from 0.0 (*t*‐test p=0.001). Despite this slight bias, the mean value is comfortably below 1.5% reported or recommended for the high dose regions by the relevant task groups.^21^, ^22^

**Figure 5:**
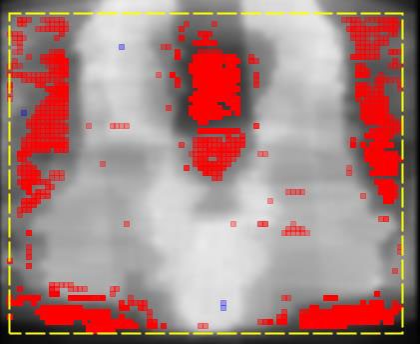
A coronal cross section for a wide‐field IMRT anal plan. The color coding over the dose‐intensity map indicates the areas in the low‐dose region that fail 3% L/2 mm gamma analysis (ACPDP vs. TPS).

The results of the direct AC to TPS comparison, and the comparison between the 3D ACPDP and TPS dose grids are presented in Table 2 and Table 3, respectively. Following the TG‐119 confidence limit methodology, for the 3% G/2 mm criteria, in 95% of the cases one should expect at least 96.3% of the points to pass with the AC and 96.9% with the ACPDP. This is above the proposed 95% investigative threshold, let alone the 90% actionable one. Moreover, the 90% pass action threshold should be exceeded or attained at the 95% confidence level even with the more stringent 2% G/2 mm criteria, for both the AC and ACPDP. Therefore it is expected that the well‐commissioned TPS, in conjunction with the new ArcCHECK hardware and software, should consistently produce gamma analysis results acceptable by the emerging standard, whether for the basic AC measurements or for 3D ACPDP on a homogeneous cylindrical phantom. At the same time, the analyses with the more sensitive local dose‐error criteria show reduced agreement, particularly with the larger targets from the TG‐244 datasets requiring wide‐field IMRT. While the average 2% L/2 ACPDP vs. TPS passing rate is a respecTable 91%, the two lowest values are 75.5% and 78.2%, for the 6X and 15X Anal wide‐field IMRT plans, respectively. These types of plans contain complex bifurcating targets separated by large low‐dose volumes and are known to be problematic in terms of dosimetric agreement, particularly in the low‐dose areas when the local dose‐error normalization is used. This is clearly illustrated in Fig. 5, where gamma analysis failures, save a few errand pixels, are confined to the areas outside the targets. Wide‐field IMRT introduces an additional challenge of modeling the leakage through the static, narrow gaps between the nearly abutting MLC leaves inside the field. Thus the TG‐244 plans serve as a useful stress test. While the ACPDP results obviously based on the AC measurements, the former shows a slightly better agreement with the TPS than the latter (Table 3 vs. Table 2), both in terms of the average gamma passing rates and average median dose‐difference across the plan population. This is consistent with the notion that the AC has an inherently challenging geometry for comparison with the TPS. Given the peripheral detector location, the AC measured dose is typically considerably lower than the central one. The 10% low dose cutoff threshold becomes perhaps 5% or so in a global sense, making it more difficult to achieve agreement with the TPS, exceedingly so with the local dose‐error normalization. The sign of the average median dose deviation (AC>TPS) suggests that further reducing the AC dose‐response nonlinearity with low MU would not improve the results, since it would increase the reported dose, if anything.

Overall, the comparison results, whether with another array dosimeter or the TPS, underscore the ultimate dilemma one faces with the intensity‐modulated dose distribution measurements: the expected accuracy of the measurement tool (∼2%) is comparable to the desired uncertainty in the measured quantity (2%–3% in dose), instead of being an order of magnitude smaller, as dictated by metrology. A relatively small systematic measurement error of say 0.5% can easily have a noticeable effect.

**Table 2:** Gamma analysis passing rates and median dose‐differences: ArcCHECK vs. TPS

		ArcCHECK vs. TPS γ *Passing Rate (%)*			
		*Local Dose‐error*	*Global Dose‐error*		
*Dataset*	*Energy/Technique*	2%/2 mm	2%/2 mm	3%/2 mm	*Median* ΔD *(%)*
Abdomen‐TG 244	6X‐VMAT	93.7	99.8	100.0	1.1
	6X‐IMRT	86.3	97.9	99.4	1.5
	15X‐VMAT	94.8	99.8	100.0	1.1
	15X‐IMRT	89.2	98.0	99.4	1.1
	10XFFF‐VMAT	98.0	100.0	100.0	‐0.2
	10XFFF‐IMRT	92.0	97.1	98.8	‐0.6
H&N‐TG 244	6X‐VMAT	86.2	98.5	100.0	1.3
	6X‐WFIMRT	78.3	95.4	98.9	2.3
	15X‐VMAT	92.0	99.8	99.9	1.4
	15X‐WFIMRT	81.8	95.8	99.0	2.3
	10XFFF‐VMAT	92.1	98.4	99.3	‐1.0
	10XFFF‐WFIMRT	90.4	97.3	99.3	0.1
Anal‐TG 244	6X‐VMAT	80.8	97.5	99.9	2.6
	6X‐WFIMRT	73.4	92.1	98.0	2.9
	15X‐VMAT	88.5	97.4	99.2	1.9
	15X‐WFIMRT	83.1	94.5	98.6	2.3
	10XFFF‐VMAT	92.1	98.9	99.6	‐0.5
	10XFFF‐WFIMRT	91.8	98.1	99.5	0.7
C‐shape‐TG 119	6X‐VMAT	80.6	93.4	97.5	2.6
	6X‐IMRT	85.4	90.4	95.3	2.4
	15X‐VMAT	86.3	95.4	98.6	1.7
	15X‐IMRT	86.5	92.1	95.6	0.8
	10XFFF‐VMAT	91.2	98.4	99.7	‐0.3
	10XFFF‐IMRT	90.2	95.2	97.2	‐0.3
H&N‐TG 119	6X‐VMAT	86.0	94.2	98.4	2.0
	6X‐IMRT	89.0	94.1	98.4	1.0
	15X‐VMAT	90.6	94.4	97.3	1.0
	15X‐IMRT	92.5	95.4	98.1	0.5
	10XFFF‐VMAT	91.2	95.3	97.4	‐0.5
	10XFFF‐IMRT	88.8	94.0	97.8	‐1.1
	Average	88.1	96.3	98.7	1.0
	SD	5.3	2.6	1.2	1.2
	Min	73.4	90.4	95.3	‐1.1
	Max	98.0	100.0	100.0	2.9

**Table 3:** Gamma analysis passing rates and median dose‐differences: ACPDP vs. TPS (on the 22 cm diameter Delta4 PMMA phantom)

		ACPDP vs. TPS γ *Passing Rate (%)*			
		*Local Dose‐error*	*Global Dose‐error*		
*Dataset*	*Energy/Technique*	2%/2 mm	2%/2 mm	3%/2 mm	*Median* ΔD *(%)*
Abdomen‐TG 244	6X‐VMAT	97.5	99.7	100.0	0.6
	6X‐IMRT	94.7	99.6	99.9	1.1
	15X‐VMAT	97.9	99.1	99.9	‐0.3
	15X‐IMRT	95.0	98.4	99.7	0.2
	10XFFF‐VMAT	98.3	99.8	100.0	‐0.6
	10XFFF‐IMRT	91.1	98.9	99.9	‐1.1
H&N‐TG 244	6X‐VMAT	90.2	98.1	99.9	0.7
	6X‐WFIMRT	83.7	93.4	99.5	1.0
	15X‐VMAT	92.2	98.7	100.0	1.0
	15X‐WFIMRT	85.2	95.8	99.4	1.6
	10XFFF‐VMAT	90.3	95.9	99.5	‐1.6
	10XFFF‐WFIMRT	91.8	97.1	99.7	‐0.9
Anal‐TG 244	6X‐VMAT	82.7	93.3	99.3	2.5
	6X‐WFIMRT	75.5	84.1	93.2	3.1
	15X‐VMAT	87.3	96.5	99.8	2.3
	15X‐WFIMRT	78.2	87.1	94.9	2.9
	10XFFF‐VMAT	95.2	99.5	99.9	‐0.5
	10XFFF‐WFIMRT	92.6	98.3	99.6	0.5
C‐shape‐TG 119	6X‐VMAT	87	96.7	99.1	2.3
	6X‐IMRT	89.6	97.5	99.3	1.5
	15X‐VMAT	91.3	98.8	99.7	1.6
	15X‐IMRT	89.3	95.9	98.5	0.9
	10XFFF‐VMAT	93.5	98.5	99.7	0.4
	10XFFF‐IMRT	93.2	98.6	99.7	0.5
H&N‐TG 119	6X‐VMAT	94.8	99.2	99.9	1.3
	6X‐IMRT	95.8	98.9	99.9	0.3
	15X‐VMAT	94.6	98.9	99.8	0.9
	15X‐IMRT	93.6	97.7	99.5	0.8
	10XFFF‐VMAT	95.3	98.6	99.7	0.1
	10XFFF‐IMRT	93.4	98.9	99.9	‐0.7
	Average	91.0	97.1	99.3	0.7
	SD	5.5	3.6	1.5	1.2
	Min	75.5	84.1	93.2	‐1.6
	Max	98.3	99.8	100.0	3.1

## IV. CONCLUSIONS

We comprehensively characterized the redesigned ArcCHECK helical dosimeter and dose reconstruction software. Previously unreported response nonlinearity with low MU settings has been quantified. Increasing the integration period compared to the current default setting is recommended to minimize the effect. While most AC parameters did not change appreciably compared to the previous versions, the out‐of‐field diode response did. The overresponse to scattered out‐of‐field radiation has decreased by as much as 17% due to the reduction of the amount of high‐Z materials in the immediate vicinity of the diodes. While objectively an improvement in the measurement accuracy, this change made the device incompatible with the existing 3D dose reconstruction software, which had to be redesigned to accommodate this and possible future changes. During the dose reconstruction software commissioning process, the user has to establish a single, energy‐independent, correction factor appropriate for the ArcCHECK hardware and software versions. After the application of such factor in this work, the accuracy of measurement‐guided dose reconstruction was restored to the previously reported level. In the process, we demonstrate with the challenging TG‐244 commissioning datasets that ACPDP vs. Pinnacle TPS gamma‐analysis passing rates on a homogeneous phantom can be expected to exceed 96% and 90% at the 95% confidence level, for the 3% G/2 mm/10% and 2% G/2 mm/10%, respectively. The local dose‐error normalization leads to wider variation in passing rates, with the larger targets presenting a challenge, as expected. Such TG‐244 datasets provide a useful stress test for both the dosimeters and the TPS. They expand the availability of publicly available, standardized commissioning/verification cases, which are more clinically relevant and in some respects more probative than those from TG‐119.

## ACKNOWLEDGMENT

This work was supported in part by a grant from Sun Nuclear Corp.

## COPYRIGHT

This work is licensed under a Creative Commons Attribution 3.0 Unported License.

## Supporting information

Supplementary Material

Supplementary Material

Supplementary Material
