# Supplementary figures and images for: Validation of an improved helical diode array and dose reconstruction software using TG‐244 datasets and stringent dose comparison criteria
Source: J Appl Clin Med Phys. 2016 Nov 8;17(6):163–78. doi: 10.1120/jacmp.v17i6.6414 (PMC5690493; doi:10.1120/jacmp.v17i6.6414)

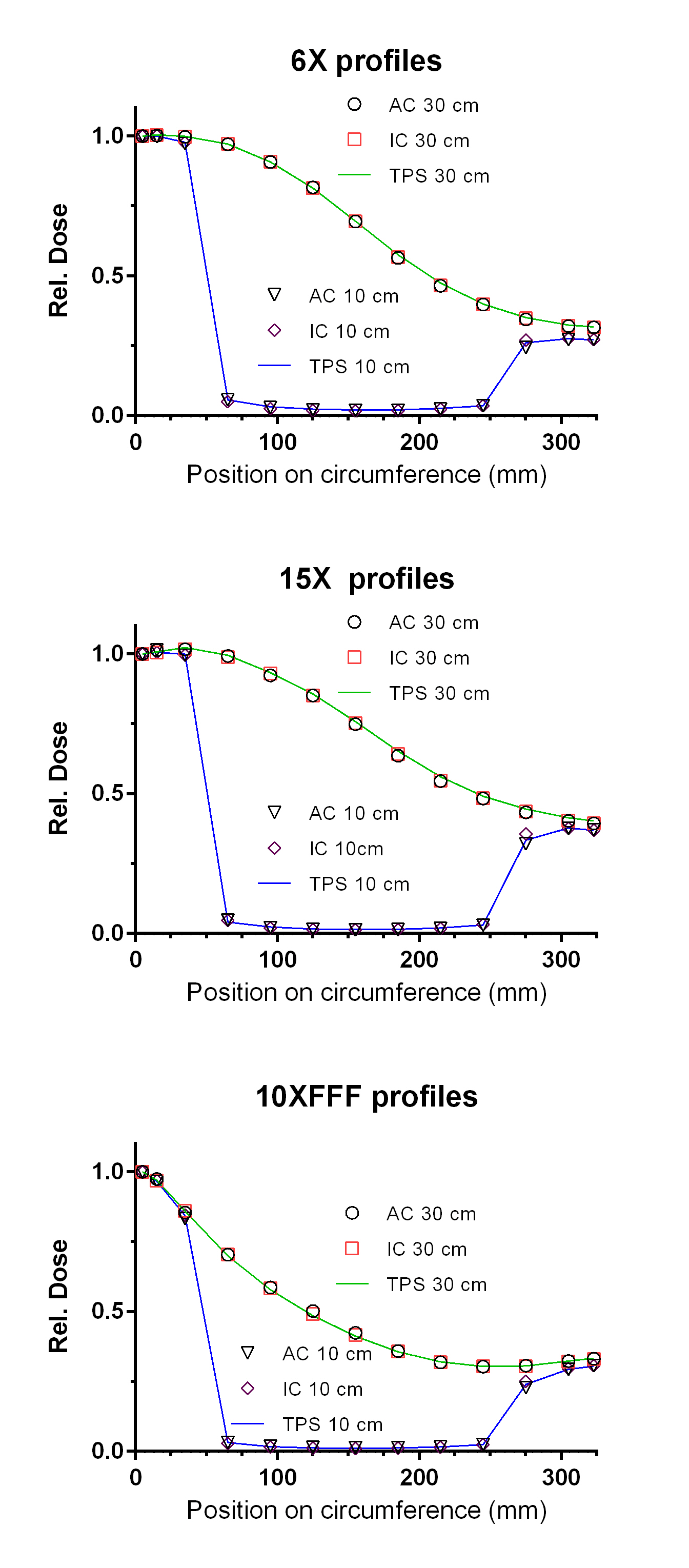

Supplement: Supplementary file 1 — Supplementary Material [file ACM2-17-163-s001.jpg]

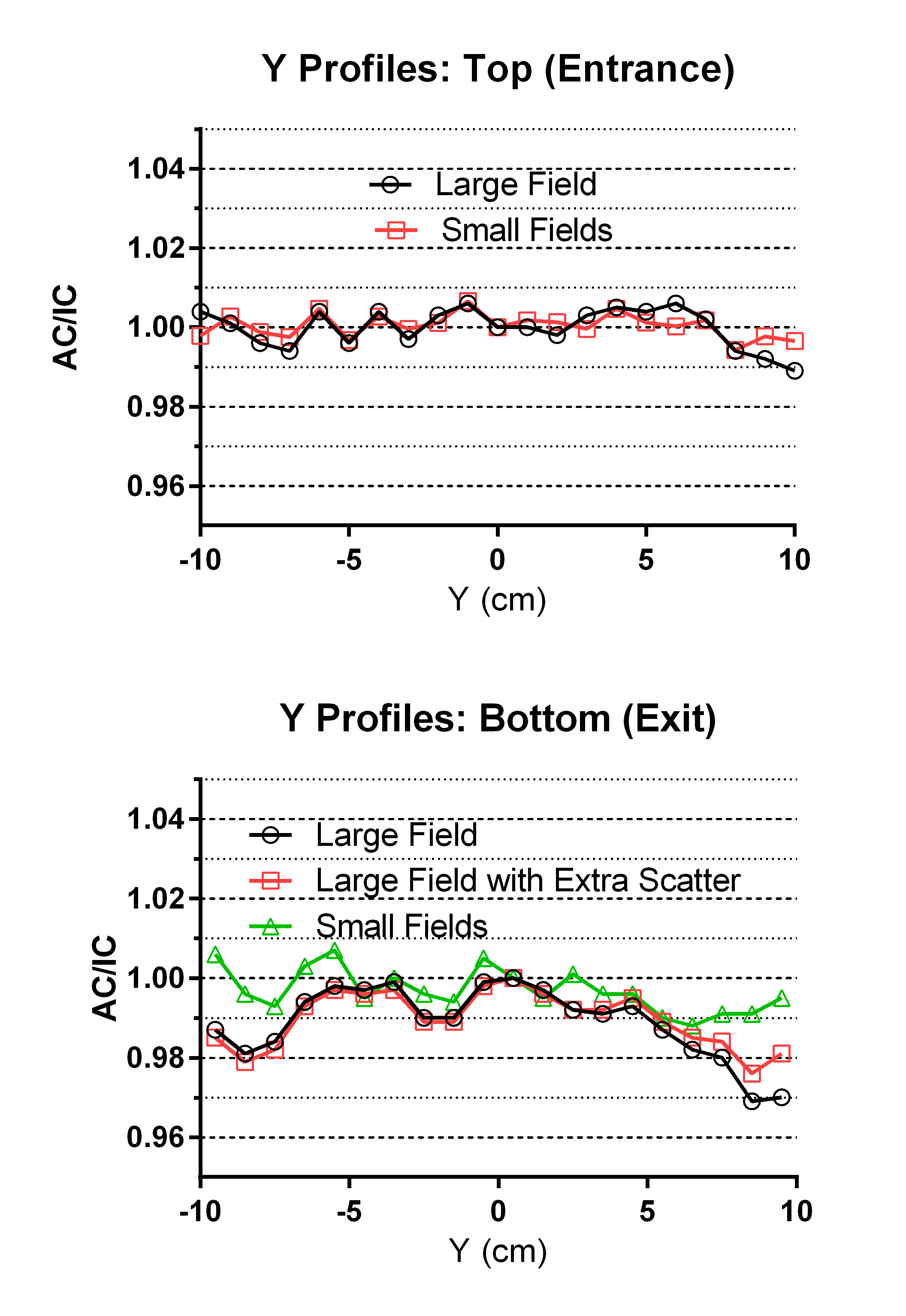

Supplement: Supplementary file 2 — Supplementary Material [file ACM2-17-163-s002.jpg]

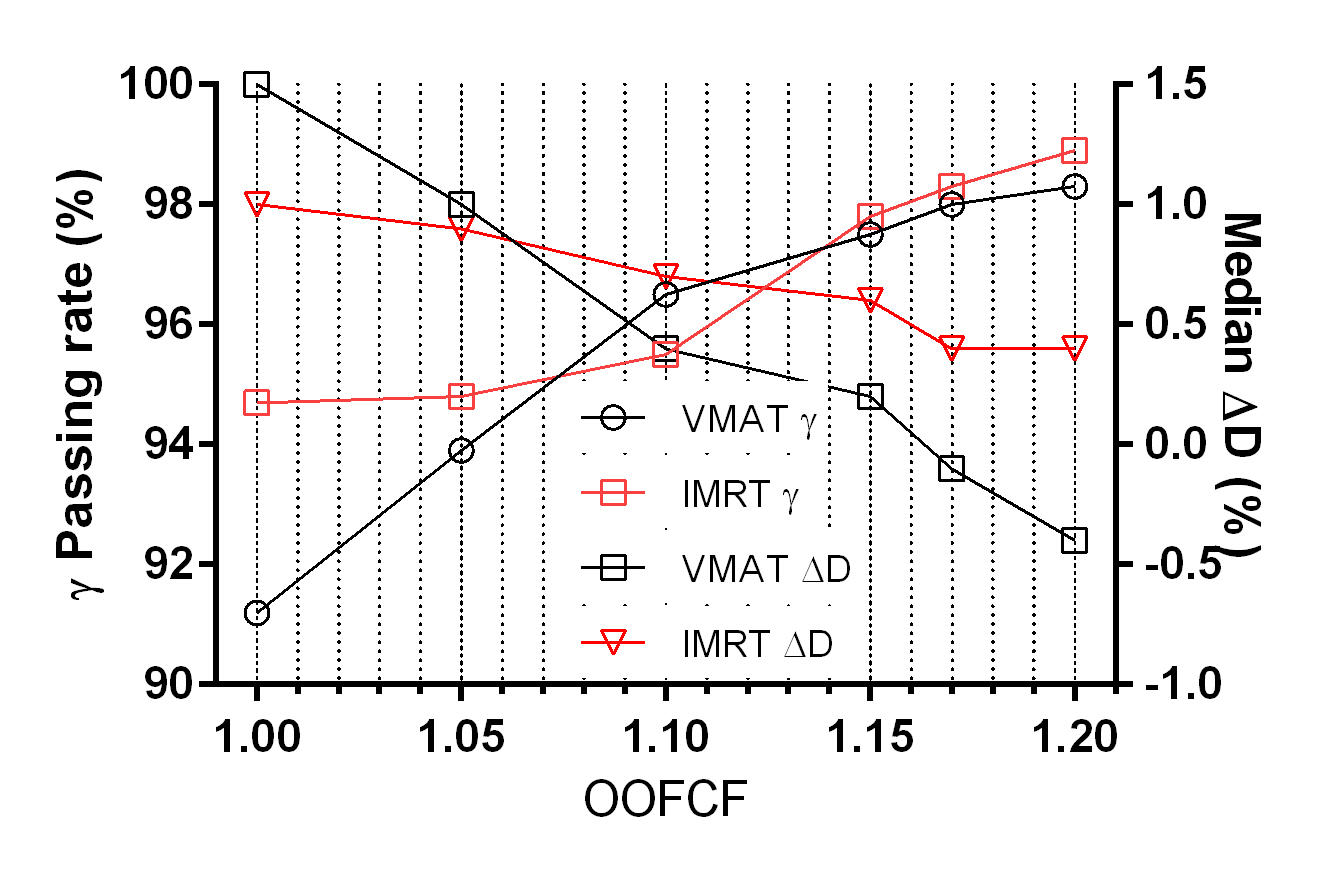

Supplement: Supplementary file 3 — Supplementary Material [file ACM2-17-163-s003.jpg]
